# Longitudinal changes in bioelectrical impedance–derived body composition after sleeve gastrectomy in patients with class III obesity

**DOI:** 10.1007/s00423-026-04232-x

**Published:** 2026-08-29

**Authors:** Lisa Lippmann, Hermann Kissler, Utz Settmacher, Falk Rauchfuss, Felix Dondorf, Oliver Rohland, Laura Schwenk, Aladdin Ali Deeb, Astrid Bauschke, Michael Ardelt

**Affiliations:** University Hospital Jena, Klinik für Allgmein-, Viszeral- und Gefäßchirurgie, Am Klinikum 1, Jena, 07747 Germany

**Keywords:** Obesity, Bariatric surgery, Sleeve gastrectomy, Bioelectrical impedance analysis, Body composition, Phase angle

## Abstract

**Purpose:**

Weight loss after bariatric surgery is commonly reported using anthropometric parameters; however, these measures do not distinguish between changes in fat mass, lean tissue, and body water. Bioelectrical impedance analysis (BIA) enables a more detailed assessment of body composition and may provide additional information regarding postoperative physiological adaptations. The present study aimed to evaluate longitudinal changes in BIA-derived body composition over a 24-month follow-up after sleeve gastrectomy (SG) in patients with class III obesity. In addition, exploratory subgroup analyses according to sex and baseline body mass index (BMI) were performed to investigate potential differences in postoperative body composition trajectories.

**Methods:**

This retrospective observational study included 49 patients who underwent laparoscopic SG at a tertiary bariatric center. Body composition was assessed preoperatively and at regular postoperative follow-up visits using standardized multifrequency BIA. Parameters included body weight, BMI, fat mass, fat-free mass, skeletal muscle mass, total body water, extracellular water-to-total body water ratio (ECW/TBW), phase angle (PhA), visceral adipose tissue estimate, and resting energy expenditure. Longitudinal changes were evaluated throughout the 24-month observation period. Subgroup analyses should be interpreted with caution because of the limited sample size.

**Results:**

Substantial reductions in body weight, BMI, fat mass, visceral adipose tissue estimate, fat-free mass, skeletal muscle mass, total body water, and estimated resting energy expenditure were observed during follow-up. The largest changes occurred during the first postoperative year and subsequently stabilized. Phase angle decreased during the early postoperative period before showing partial recovery over time. Exploratory subgroup analyses suggested differences in absolute body composition trajectories between men and women as well as between patients with baseline BMI below and above 50 kg/m²; however, these findings should be interpreted within the context of the observational study design and limited statistical power.

**Conclusion:**

Sleeve gastrectomy was associated with marked longitudinal changes in BIA-derived estimates of body composition during the first 24 postoperative months. Besides the expected reduction in fat mass, decreases in fat-free mass and skeletal muscle mass as well as transient changes in phase angle were observed. These findings emphasize the importance of longitudinal body composition assessment following bariatric surgery. Future studies should investigate whether structured nutritional and exercise interventions may further improve postoperative body composition outcomes. Because body composition was assessed exclusively by BIA, the results should be interpreted as impedance-derived estimates rather than direct measurements of individual tissue compartments.

## Introduction

The prevalence of obesity has risen steadily worldwide in recent decades. Between 1980 and 2015, it doubled in more than 70 countries and continued to increase in most other countries [1]. Even a body mass index (BMI) above the normal range significantly increases the risk of comorbidities such as cardiovascular disease or diabetes mellitus [2]. Obesity affects almost every organ system and is associated with hypertension, dyslipidemia, insulin resistance, type 2 diabetes, liver disease, malignant diseases, and psychological stress [3].

Metabolic and bariatric surgery is an established treatment for severe obesity. Contemporary guidance emphasizes BMI, metabolic disease, individualized risk-benefit assessment, and multidisciplinary perioperative evaluation rather than a single prescribed duration of unsuccessful conservative therapy [4, 5]. Alongside Roux-en-Y gastric bypass, sleeve gastrectomy (SG) is an effective procedure and commonly produces total weight loss of approximately 25%-30% during the first postoperative year [6, 7]. However, weight loss is not restricted to fat mass and may include substantial changes in lean tissue and body-water compartments that are not captured by body weight or BMI alone [8].

Bioelectrical impedance analysis (BIA) provides rapid, non-invasive estimates of fat mass, fat-free mass, skeletal muscle mass, and body-water compartments and is therefore suitable for serial postoperative assessment. Phase angle (PhA), calculated directly from resistance and reactance [arctangent (reactance/resistance) x 180/pi], reflects the relative contribution of tissue reactance and resistance and has been used as a composite impedance-derived marker of body cell mass, hydration, and nutritional status rather than as a direct measure of cellular integrity [9, 10]. After bariatric surgery, the greatest loss of fat-free or lean mass generally occurs during the first postoperative months, and systematic reviews have also described an early decrease in PhA [11–14]. The primary aim of this study was to describe longitudinal changes in BIA-derived body composition over 24 months after SG. Secondary exploratory analyses assessed whether postoperative trajectories differed by sex or by baseline BMI (< 50 vs. ≥50 kg/m²).

## Materials and methods

### Study design

This retrospective observational study included 49 consecutive patients (36 women and 13 men) who underwent laparoscopic sleeve gastrectomy (SG) at the Department of General, Visceral and Vascular Surgery, Jena University Hospital, between January 2019 and September 2021. Patients were eligible if they were at least 18 years of age, fulfilled the World Health Organization criteria for class III obesity (BMI ≥ 40 kg/m²), and completed the standardized postoperative follow-up protocol including repeated bioelectrical impedance analysis (BIA) measurements. Patients with hyperthyroidism, Cushing’s disease, active malignant disease, severe inflammatory disorders, or missing consent for the scientific use of anonymized clinical data were excluded.

The primary objective of this study was to describe longitudinal changes in BIA-derived body composition following SG over a 24-month follow-up period. In addition, exploratory subgroup analyses according to sex and baseline BMI (< 50 vs. ≥50 kg/m²) were performed to evaluate potential differences in postoperative body composition trajectories. Sex was selected because of established differences in lean mass and adipose-tissue distribution [15, 16], while the BMI threshold of 50 kg/m² was selected a priori as a conventional clinical boundary for very severe obesity. Because of the limited sample size, these subgroup analyses were considered hypothesis-generating rather than confirmatory.

Clinical and body composition data were collected retrospectively from the institutional bariatric database. Patients underwent routine clinical follow-up preoperatively and approximately 3, 6, 9, 12, 18, and 24 months after surgery according to the standardized follow-up schedule (see Table 1).

**Table 1:** Baseline body composition characteristics of patients before SG, stratified by sex

	All patients, *n* = 49 [25–75]	Male, *n* = 13 [25–75]	Female, *n* = 36 [25–75]	*P*-value
BMI (kg/m²)	47.40 [42.85, 53.17]	46.06 [39.51, 53.17]	47.48 [43.25, 53.86]	0.618
Weight (kg)	130.00 [118.00, 150.00]	145.00 [125.5, 179.5]	127.5 [115, 144.5]	0.025
Absolute fat mass (kg)	67.08 [59.49, 78.69]	62.99 [49.68, 80.76]	67.27 [59.89, 75.78]	0.667
Visceral adipose tissue (L)	7.95 [5.56, 11.19]	12.54 [10.36, 15.81]	6.62 [5.17, 8.93]	< 0.001
Fat-free mass (kg)	62.97 [54.36, 73.61]	82.24 [72.82, 95.24]	59.19 [53.19, 65.57]	< 0.001
Skeletal muscle mass (kg)	31.23	39.98 [34.97, 49.69]	30.35 [26.78, 33.09]	< 0.001
Total body water (L)	48.30 [41.53, 56.09]	61.71 [54.26, 71.51]	45.43 [40.64, 50.44]	< 0.001
ECW/TBW (%)	45.21 [44.43, 46.87]	44.3 [43.75, 44.64]	45.87 [45.0, 47.2]	< 0.001
Phase angle (°)	5.85 [5.53, 6.41]	5.58 [5.42, 5.88]	6.06 [5.6, 6.5]	0.054
SDS phase angle (z-score)	0.00 [–1.14, 0.84]	-1.85 [(-2.35), (-1.14)]	0.49 (-0.24, 1.02)	< 0.001
Phase angle percentile (%)	50.00 [10.50, 80.00]	3.0 [1.0, 13.0]	69.0 [30.0, 89.25]	< 0.001
Resting energy expenditure (kcal/day)	2159.36 [1975.85, 2404.92]	2486.78 [2223.43, 2925.6]	2023.06 [1893.74, 2224.18]	< 0.001

Data are presented as median [interquartile range, 25th–75th]. Comparisons between male and female patients were performed using the Mann–Whitney U test (*p*-values)

*BMI* body mass index, *ECW/TBW* ratio of extracellular water to total body water, *SDS* phase angle, standardized deviation score of phase angle

A formal a priori sample size calculation was not performed because this investigation was based on a predefined retrospective cohort. Consequently, the study was not specifically powered for subgroup analyses, and both type I and type II statistical errors cannot be excluded. These aspects should be considered when interpreting subgroup findings and are addressed in the Limitations section.

### Anthropometric assessment

Body weight (BW), height, and body mass index (BMI) were recorded at every study visit using standardized clinical procedures. Body weight was measured to the nearest 0.1 kg using calibrated digital scales, while height was measured without shoes using a wall-mounted stadiometer. BMI was calculated as body weight divided by height squared (kg/m²).

Percentage total weight loss (%TWL) and percentage excess weight loss (%EWL) were calculated using the institutional standard formulas. These parameters were included because they represent the most commonly reported clinical outcome measures after bariatric surgery and facilitate comparison with previous studies. Nevertheless, they do not fully reflect postoperative alterations in body composition.

### Bioelectrical impedance analysis

Body composition was assessed using a multifrequency segmental bioelectrical impedance analyzer (seca mBCA 515, seca GmbH & Co. KG, Hamburg, Germany). All measurements were performed according to the manufacturer’s standardized recommendations to minimize measurement variability. Participants were examined in the morning after an overnight fast, following bladder emptying, without recent vigorous physical activity, and while standing barefoot on the measurement platform holding the hand electrodes.

The BIA device automatically generated estimates of fat mass (FM), fat-free mass (FFM), skeletal muscle mass (SMM), total body water (TBW), extracellular water (ECW), extracellular water-to-total body water ratio (ECW/TBW), visceral adipose tissue, resting energy expenditure (REE), and phase angle (PhA). Resistance and reactance were measured directly by the device, whereas the remaining body composition parameters were calculated using manufacturer-specific prediction equations.

Phase angle was derived from the relationship between electrical resistance and reactance and represents an impedance-derived parameter reflecting the relative contribution of tissue resistance and reactance. Because resistance is strongly influenced by body water and reactance is related to the capacitive properties of biological tissues, PhA has been proposed as an indirect marker of nutritional status and body cell mass. However, it should not be interpreted as a direct measure of cellular integrity or muscle quality.

No post-hoc correction or recalibration of the manufacturer-derived body composition estimates was performed.

### Statistical analysis

Statistical analyses were performed using IBM SPSS Statistics (Version 29.0; IBM Corp., Armonk, NY, USA). Continuous variables are presented as median and interquartile range unless otherwise stated. Longitudinal changes during follow-up were analyzed using linear mixed models to account for repeated measurements within individuals and to allow inclusion of all available observations despite incomplete follow-up. This approach reduces the loss of information associated with complete-case analyses and is considered appropriate for longitudinal observational data.

Time was included as a fixed effect, while individual patients were treated as random effects. Secondary analyses investigated potential interactions between time and sex as well as between time and baseline BMI category (< 50 vs. ≥50 kg/m²). Because these subgroup analyses were exploratory and the study cohort was relatively small, the corresponding results should be interpreted cautiously.

No formal adjustment for multiple statistical testing was performed. Therefore, subgroup analyses and individual comparisons should primarily be regarded as exploratory and hypothesis-generating rather than definitive evidence. Emphasis was placed on the overall longitudinal patterns and the consistency of the observed findings rather than isolated statistically significant results. Statistical significance was defined as a two-sided p value < 0.05.

## Results

### Patient characteristics

A total of 49 patients were included in the present study, comprising 36 women (73.5%) and 13 men (26.5%). Baseline demographic and body composition characteristics are summarized in Tables 1 and 2. Median preoperative BMI indicated severe obesity in the overall study population, while men demonstrated significantly higher absolute body weight, fat-free mass, skeletal muscle mass, total body water, visceral adipose tissue estimate, and estimated resting energy expenditure than women (see Table 1). In contrast, baseline BMI did not differ significantly between sexes.

**Table 2:** Baseline body composition characteristics of patients before SG, stratified by BMI category

	All patients, *n* = 49 [25–75]	BMI < 50, *n* = 29 [25–75]	BMI ≥ 50, *n* = 20 [25–75]	*P*-value
BMI (kg/m²)	47.40 [42.94, 52.80]	43.25 [39.71, 45.44]	54.23 [52.01, 56.88]	< 0.001
Weight (kg)	130.00 [118.00, 149.00]	128.00 [118.00, 149.00]	134.50 [121.75, 148.00]	0.569
Absolute fat mass (kg)	67.08 [59.68, 78.63]	64.96 [59.68, 77.00]	68.87 [61.21, 78.66]	0.815
Visceral adipose tissue (L)	7.95 [5.61, 10.97]	6.71 [5.10, 10.92]	8.22 [6.56, 11.15]	0.36
Fat-free mass (kg)	62.97 [54.88, 73.30]	62.92 [53.84, 69.12]	65.36 [57.74, 76.48]	0.47
Skeletal muscle mass (kg)	31.23 [27.94, 36.43]	30.48 [27.25, 35.31]	32.81 [29.93, 38.13]	0.31
Total body water (L)	48.30	48.05 [41.27, 53.11]	49.84 [44.30, 57.41]	0.47
ECW/TBW (%)	45.21 [44.43, 46.86]	45.08	45.73 [44.65, 46.29]	0.496
Phase angle (°)	5.85 [5.55, 6.40]	5.91 [5.36, 6.41]	5.83 [5.61, 6.21]	0.903
SDS phase angle (z-score)	0.00 [–1.13, 0.83]	0.00 [–1.14, 0.89]	–0.04 [–1.02, 0.76]	0.823
Phase angle percentile (%)	50.00 [13.00, 80.00]	50.00 [5.00, 81.00]	48.50 [15.25, 78.00]	1.00
Resting energy expenditure (kcal/day)	2159.36 [1977.43, 2351.72]	2051.49 [1974.26, 2458.12]	2160.23 [2018.54, 2345.03]	0.63

Data are presented as median [interquartile range, 25th–75th]. Comparisons between BMI groups (< 50 vs. ≥ 50 kg/m²) were performed using the Mann–Whitney U test (*p*-values)

*BMI* body mass index, *ECW/TBW* ratio of extracellular water to total body water, *SDS* phase angle, standardized deviation score of phase angle

When patients were stratified according to baseline BMI (< 50 vs. ≥50 kg/m²), significant differences were observed across most absolute body composition parameters, as expected from differences in baseline body size (see Table 2). In contrast, phase angle did not differ significantly between the BMI groups.

### Longitudinal changes in body weight and BMI

Body weight and BMI decreased continuously following sleeve gastrectomy throughout the observation period (see Table 3). The most pronounced reductions occurred during the first postoperative year, whereas weight loss tended to stabilize between 12 and 24 months. This overall pattern was observed in both sexes and in both BMI groups.

**Table 3:** Changes in BMI, weight, %TWL, and %EWL during the course of follow-up

	Pre-OP	3 M	6 M	9 M	12 M	18 M	24 M	*P*-value
BMI (kg/m²)	47.4 [42.94, 52.80]	39.9 [36.51, 44.62]	36.3 [32.41, 39.10]	33.5 [31.04, 37.27]	31.9 [29.38, 36.65]	31.6 [27.80, 35.93]	32.5 [28.67, 36.81]	< 0.001
Weight (kg)	130.0 [118.00, 149.00]	112.5 [100.30, 129.40]	103.7 [92.00, 115.00]	94.5 [88.25, 105.25]	91.0 [85.00, 98.50]	89.5 [78.40, 95.00]	90.0 [81.50, 99.55]	< 0.001
%TWL (%)		13.2 [11.02, 16.18]	22.7 [19.70, 27.11]	29.4 [23.52, 35.95]	31.8 [22.91, 38.69]	32.9 [24.32, 39.12]	30.1 [21.03, 35.98]	< 0.001
%EWL (%)		31.1 [23.15, 37.01]	50.9 [40.76, 60.72]	63.2 [47.62, 72.48]	65.9 [51.39, 82.06]	68.4 [54.04, 89.30]	66.6 [49.59, 82.24]	< 0.001

Descriptive data are presented as median [interquartile range]. The *p*-values for the evaluation of BMI, weight, %TWL, and %EWL were determined using the Kruskal–Wallis test for time-dependent changes

*Pre-OP* preoperative, *M* month, *BMI* body mass index, *%TWL* percentage total weight loss, *%EWL* percentage excess weight loss

Men demonstrated larger absolute reductions in body weight than women during follow-up, reflecting their higher baseline body weight. Similarly, patients with baseline BMI ≥ 50 kg/m² experienced greater absolute weight reduction than patients with BMI < 50 kg/m². However, these findings should primarily be interpreted in relation to the marked baseline differences between the respective groups rather than as evidence of different biological responses to surgery.

Overall, sleeve gastrectomy resulted in a marked and sustained reduction in anthropometric parameters throughout the 24-month follow-up period (see Table 3).

### Percentage total weight loss and excess weight loss

Percentage total weight loss (%TWL) and percentage excess weight loss (%EWL) increased rapidly during the first postoperative months and reached their highest values approximately 18 months after surgery before remaining largely stable until the end of follow-up (see Table 3).

The temporal pattern of %TWL and %EWL closely reflected the changes observed for absolute body weight. While absolute weight loss differed between BMI groups because of baseline body size, relative postoperative weight loss demonstrated a more homogeneous pattern across the study population. No clinically relevant differences between women and men were observed regarding %TWL or %EWL.

These findings indicate that SG resulted in substantial and sustained weight reduction over the entire observation period.

### Fat mass and visceral adipose tissue

Fat mass decreased continuously following surgery and represented the largest component of overall weight reduction (see Table 4). The greatest decline was observed during the first postoperative year, followed by a slower reduction thereafter. In the longitudinal mixed model, fat mass decreased by 34.97 kg (95% CI 32.05–37.89) at 12 months and by 34.29 kg (95% CI 31.19–37.38) at 24 months compared with baseline. These decreases represented approximately 80.9% and 80.8% of the corresponding model-estimated total weight loss, respectively. This pattern was consistent across all study groups. Additional sex- and BMI-stratified trajectories are presented in Tables 6 and 7.

**Table 4:** Changes in BMI, weight, %TWL and %EWL during follow-up, stratified by sex

Parameter	Group	Pre-OP	3 M	6 M	9 M	12 M	18 M	24 M	
BMI (kg/m²)	Male	46.1 [39.7, 52.8]	36.5 [34.4, 41.6]	34.2 [31.4, 36.8]	31.6 [30.2, 34.2]	30.7 [28.3, 31.9]	30.3 [27.9, 31.3]	30.4 [28.7, 32.2]	0.002
	Female	47.5 [43.3, 52.7]	41.0 [37.4, 46.9]	38.3 [32.9, 40.6]	33.8 [31.5, 38.3]	34.2 [29.7, 37.1]	32.2 [27.8, 36.6]	33.3 [28.7, 37.5]	< 0.001
Weight (kg)	Male	145.0 [128.0, 175.0]	114.5 [107.5, 132.5]	105.5 [101.8, 118.6]	101.3 [95.2, 108.6]	95.1 [90.0, 101.0]	94.0 [88.8, 100.2]	94.5 [89.2, 104.5]	0.002
	Female	127.5 [115.0, 141.5]	110.7 [100.0, 122.0]	103.0 [87.0, 113.0]	91.8 [84.8, 96.7]	90.0 [82.2, 95.4]	87.0 [75.0, 95.0]	88.0 [79.0, 99.0]	< 0.001
%TWL (%)	Male	0.0 [0.0, 0.0]	15.6 [13.4, 21.6]	21.7 [19.7, 27.8]	31.5 [23.3, 36.8]	33.1 [26.8, 40.2]	38.5 [26.7, 46.7]	36.0 [30.5, 39.6]	0.002
	Female	0.0 [0.0, 0.0]	12.4 [10.7, 15.9]	23.8 [19.7, 27.1]	28.8 [24.0, 34.8]	30.9 [22.3, 35.6]	30.3 [24.3, 35.1]	28.8 [20.8, 32.6]	< 0.001
%EWL (%)	Male	0.0 [0.0, 0.0]	35.9 [33.0, 45.1]	56.0 [49.4, 63.3]	69.4 [60.9, 82.4]	77.0 [61.2, 85.1]	75.3 [66.7, 90.8]	78.9 [60.9, 86.8]	0.002
	Female	0.0 [0.0, 0.0]	27.1 [21.2, 32.9]	49.8 [39.0, 56.6]	58.8 [45.2, 68.3]	62.3 [50.0, 80.8]	67.4 [51.6, 87.9]	62.9 [46.8, 76.8]	< 0.001

Descriptive data are presented as median [interquartile range]. *P*-values represent time-dependent changes within each sex and were calculated using the Friedman test

*Pre-OP* preoperative, *M* month, *BMI* body mass index, *%TWL* percentage total weight loss, *%EWL* percentage excess weight loss

The estimated visceral adipose tissue volume also decreased markedly during follow-up (see Table 4). Men exhibited higher baseline visceral adipose tissue estimates than women, and patients with BMI ≥ 50 kg/m² demonstrated higher values than those with BMI < 50 kg/m². Consequently, these groups also showed larger absolute reductions throughout follow-up. The corresponding subgroup trajectories by sex and baseline BMI are shown in Tables 6 and 7.

Because visceral adipose tissue was estimated using manufacturer-specific BIA algorithms rather than direct imaging techniques, these findings should be interpreted as longitudinal changes in BIA-derived estimates rather than direct measurements of visceral fat volume. Nevertheless, the overall trend was consistent throughout the observation period.

### Fat-free mass and skeletal muscle mass

Fat-free mass and skeletal muscle mass decreased significantly following sleeve gastrectomy (see Table 5). Similar to body weight and fat mass, the most pronounced reductions occurred during the first postoperative year, whereas subsequent changes were comparatively small. In the longitudinal mixed model, fat-free mass decreased by 8.25 kg (95% CI 6.87–9.62) at 12 months and by 8.17 kg (95% CI 6.71–9.63) at 24 months compared with baseline. Relative to the corresponding model-estimated total weight decreases of 43.20 kg and 42.44 kg, fat-free mass accounted for approximately 19.1% and 19.3% of total weight loss at 12 and 24 months, respectively Sex- and BMI-stratified changes in fat-free mass and skeletal muscle mass are presented in Tables 8 and 9.

**Table 5:** Changes in BMI, weight, %TWL, and %EWL during follow-up, stratified by BMI category (< 50 vs. ≥ 50 kg/m²)

Parameter	Group	Pre-OP	3 M	6 M	9 M	12 M	18 M	24 M	
BMI (kg/m²)	BMI < 50	43.3 [39.7, 45.4]	38.0 [34.4, 41.6]	35.4 [32.0, 37.4]	32.4 [30.8, 34.6]	31.6 [29.5, 33.6]	30.8 [28.7, 33.2]	31.3 [28.7, 33.7]	< 0.001
	BMI ≥ 50	54.2 [52.0, 56.9]	41.0 [37.9, 46.9]	37.8 [33.5, 40.6]	35.3 [31.5, 38.3]	34.2 [30.6, 37.1]	32.7 [27.8, 36.6]	33.8 [28.7, 37.5]	< 0.001
Weight (kg)	BMI < 50	128.0 [118.0, 149.0]	109.5 [100.3, 121.8]	101.5 [92.0, 110.5]	93.0 [88.3, 99.5]	90.0 [85.0, 95.4]	89.0 [78.4, 95.0]	89.5 [81.5, 99.6]	< 0.001
	BMI ≥ 50	134.5 [121.8, 148.0]	115.0 [107.5, 132.5]	106.0 [101.8, 118.6]	101.3 [95.2, 108.6]	95.1	94.0 [88.8, 100.2]	94.5 [89.2, 104.5]	< 0.001
%TWL (%)	BMI < 50	0.0	13.0 [10.7, 15.9]	21.5 [19.5, 24.5]	26.9 [22.2, 30.9]	25.7 [21.8, 34.5]	29.5 [21.4, 33.8]	28.7 [19.7, 31.9]	< 0.001
	BMI ≥ 50	0.0	15.2 [11.9, 17.0]	25.6 [21.2, 29.7]	34.7 [25.3, 36.4]	36.3 [29.5, 42.0]	38.5 [29.7, 46.7]	32.6 [29.8, 44.8]	< 0.001
%EWL (%)	BMI < 50	0.0	31.5 [23.9, 41.9]	54.4 [45.8, 61.8]	64.2 [51.0, 77.7]	67.3 [51.4, 84.2]	67.7 [54.0, 84.5]	67.4 [46.3, 81.3]	< 0.001
	BMI ≥ 50	0.0	27.1 [22.2, 32.9]	49.8 [38.9, 55.5]	62.3 [46.5, 70.3]	64.3 [55.0, 81.6]	70.1 [56.0, 89.6]	62.9 [53.2, 81.5]	< 0.001

Descriptive data are presented as median [interquartile range]. *P*-values represent time-dependent changes within each BMI category and were calculated using the Friedman test

*Pre-OP* preoperative, *M* month, *BMI* body mass index, *%TWL* percentage total weight loss, *%EWL* percentage excess weight loss

**Table 6:** Changes in fat mass and visceral adipose tissue during follow-up, stratified by sex

Parameter	Group	Pre-OP	3 M	6 M	9 M	12 M	18 M	24 M	
Absolute fat mass (kg)	Male	63.0 [52.1, 72.4]	46.7 [40.5, 59.4]	37.7 [30.3, 47.8]	29.5 [26.1, 34.8]	24.2 [16.6, 27.4]	24.1 [19.0, 27.6]	21.3 [18.6, 31.2]	< 0.001
	Female	67.3 [60.6, 78.7]	57.3 [49.1, 70.3]	46.3 [38.8, 55.5]	38.9 [35.4, 46.8]	38.6 [29.4, 42.3]	34.4 [26.5, 42.9]	36.8 [26.7, 44.1]	< 0.001
Visceral adipose tissue	Male	12.5 [11.0, 14.8]	8.8 [7.0, 12.3]	6.1 [4.9, 9.6]	5.1 [4.2, 6.9]	3.6 [2.8, 5.4]	4.1 [3.4, 4.6]	4.2 [2.5, 5.4]	0.001
	Female	6.6 [5.3, 8.9]	4.4 [3.6, 6.2]	3.1 [2.5, 5.3]	2.2 [2.0, 3.4]	2.2 [0.9, 2.9]	1.9 [1.2, 3.1]	2.2 [1.2, 3.3]	< 0.001

Descriptive data are presented as median [interquartile range]. *P*-values represent time-dependent changes within each sex and were calculated using the Friedman test

*Pre-OP* preoperative, *M* month

**Table 7:** Changes in fat mass and visceral adipose tissue during follow-up, stratified by BMI category (< 50 vs. ≥ 50 kg/m²)

Parameter	Group	Pre-OP	3 M	6 M	9 M	12 M	18 M	24 M	
Absolute fat mass (kg)	BMI < 50	61.2 [50.2, 64.0]	48.3 [41.6, 51.7]	36.8 [32.2, 43.2]	34.0 [26.2, 39.0]	33.1 [22.4, 40.3]	31.8 [25.5, 39.8]	31.9 [23.8, 39.0]	< 0.001
	BMI ≥ 50	78.8 [74.1, 89.5]	71.6 [68.9, 73.4]	54.5 [51.0, 60.4]	41.6 [35.0, 48.9]	34.2 [28.2, 46.3]	27.6 [23.0, 43.8]	38.4 [22.2, 45.3]	< 0.001
Visceral adipose tissue	BMI < 50	5.8 [4.8, 8.0]	4.3 [3.3, 5.8]	3.0 [2.4, 5.2]	2.1 [1.9, 2.9]	2.2 [1.2, 3.0]	2.4 [1.3, 3.5]	1.9 [1.1, 2.8]	< 0.001
	BMI ≥ 50	10.8 [8.8, 14.5]	8.4 [6.2, 10.3]	5.7 [4.0, 7.5]	4.4 [2.8, 5.1]	3.0 [2.0, 4.3]	2.8 [1.6, 3.4]	3.5 [2.4, 4.6]	< 0.001

Descriptive data are presented as median [interquartile range]. *P*-values represent time-dependent changes within each BMI category and were calculated using the Friedman test

*Pre-OP* preoperative, *M* month, *BMI* body mass index

**Table 8:** Changes in fat mass and visceral adipose tissue during follow-up, stratified by sex

Parameter	Group	Pre-OP	3 M	6 M	9 M	12 M	18 M	24 M	
Fat-free mass (kg)	Male	82.2 [73.9, 94.9]	68.6 [65.5, 78.7]	71.5 [61.8, 78.5]	71.9 [63.5, 81.2]	70.9 [67.0, 81.0]	68.6 [63.6, 71.9]	72.1 [64.0, 79.7]	0.053
	Female	59.2 [53.3, 64.3]	51.2 [48.5, 58.4]	53.2 [48.2, 57.2]	50.2 [48.1, 58.5]	51.4 [49.5, 56.2]	51.7 [47.8, 57.7]	51.9 [48.7, 59.7]	< 0.001
Skeletal muscle mass (kg)	Male	40.0 [35.0, 49.1]	32.1 [31.4, 38.5]	33.3 [28.8, 36.8]	32.8 [28.8, 38.3]	33.6 [30.5, 37.3]	31.6 [30.3, 33.3]	33.7 [30.0, 38.2]	0.014
	Female	30.3 [27.0, 32.5]	25.0 [23.1, 27.9]	24.0 [21.2, 26.8]	23.1 [21.5, 26.7]	24.0 [21.4, 25.9]	23.1 [21.0, 27.2]	23.4 [21.7, 28.6]	< 0.001

Descriptive data are presented as median [interquartile range]. *P*-values represent time-dependent changes within each sex and were calculated using the Friedman test

*Pre-OP* preoperative, *M* month

**Table 9:** Changes in fat-free mass and skeletal muscle mass during follow-up, stratified by BMI category (< 50 vs. ≥ 50 kg/m²)

Parameter	Group	Pre-OP	3 M	6 M	9 M	12 M	18 M	24 M	
Fat-free mass (kg)	BMI < 50	57.8 [53.3, 67.0]	55.5 [49.4, 61.0]	56.1 [48.1, 61.4]	53.4 [49.9, 61.1]	52.4 [50.0, 62.5]	52.1 [48.0, 60.3]	52.2 [49.3, 60.7]	0.004
	BMI ≥ 50	70.8 [62.8, 86.3]	58.8 [50.9, 65.3]	59.7 [53.5, 75.5]	61.7 [48.2, 78.0]	56.3 [51.8, 73.7]	59.8 [51.1, 70.7]	61.8 [53.0, 74.3]	0.002
Skeletal muscle mass (kg)	BMI < 50	29.2 [27.2, 32.8]	25.4 [23.5, 29.4]	26.1 [21.9, 28.8]	24.6 [22.8, 28.5]	24.5 [21.8, 29.3]	25.0 [21.8, 27.9]	24.6 [22.3, 28.7]	< 0.001
	BMI ≥ 50	35.9 [31.1, 44.4]	28.2 [25.0, 33.1]	28.0 [24.0, 36.4]	28.0 [22.3, 35.6]	26.1 [22.6, 34.1]	27.7 [22.7, 32.1]	29.9 [23.6, 35.0]	< 0.001

Descriptive data are presented as median [interquartile range]. *P*-values represent time-dependent changes within each BMI category and were calculated using the Friedman test

*Pre-OP* preoperative, *M* month, *BMI* body mass index

**Table 10:** Changes in body water distribution during follow-up, stratified by sex

Parameter		Pre-OP	3 M	6 M	9 M	12 M	18 M	24 M	
Total body water (L)	Male	61.7 [55.1, 71.5]	51.3 [49.0, 59.0]	53.4 [46.1, 58.3]	53.4 [47.3, 60.4]	52.6 [49.6, 60.0]	50.8 [47.0, 53.2]	53.1 [47.4, 58.9]	0.056
	Female	45.4 [40.7, 49.2]	39.1 [36.8, 45.2]	40.3 [36.8, 43.5]	38.1 [36.6, 44.4]	38.8 [37.4, 43.0]	39.1 [36.1, 43.5]	39.4 [36.7, 45.2]	< 0.001
ECW/TBW (%)	Male	44.4 [43.8, 44.6]	44.7 [44.4, 45.5]	45.3 [44.9, 46.1]	45.4 [44.6, 45.7]	45.0 [44.3, 45.9]	44.9 [44.4, 45.8]	44.4 [44.1, 44.8]	0.074
	Female	45.9	47.1 [45.7, 48.2]	47.4 [46.8, 48.3]	47.7 [46.7, 48.3]	47.4 [46.4, 48.2]	47.2 [46.2, 48.1]	47.0 [45.5, 48.5]	0.02

Descriptive data are presented as median [interquartile range]. *P*-values represent time-dependent changes within each sex and were calculated using the Friedman test

*Pre-OP* preoperative, *M* month, *ECW* extracellular water, *TBW* total body water

**Table 11:** Changes in body water distribution during follow-up, stratified by BMI category (< 50 vs. ≥ 50 kg/m²)

Parameter		Pre-OP	3 M	6 M	9 M	12 M	18 M	24 M	
Total body water (L)	BMI < 50	44.3 [40.7, 50.3]	42.2 [37.6, 46.4]	42.5 [36.6, 45.7]	40.4 [37.8, 46.1]	39.8 [37.6, 46.8]	39.5 [36.6, 45.6]	39.7 [37.2, 46.1]	0.001
	BMI ≥ 50	54.4 [48.2, 65.3]	45.5 [39.1, 49.9]	45.8 [40.9, 56.5]	46.7 [36.8, 57.8]	43.0 [39.4, 54.9]	45.0 [38.7, 52.4]	47.0 [40.3, 54.8]	0.001
ECW/TBW (%)	BMI < 50	45.1 [44.4, 46.0]	46.0 [45.3, 47.6]	46.4 [45.6, 47.3]	46.2 [45.4, 47.7]	46.4 [45.5, 47.6]	46.4 [45.5, 47.5]	46.3 [44.9, 47.8]	0.039
	BMI ≥ 50	45.4 [44.5, 47.3]	46.1 [44.9, 48.0]	47.0 [45.3, 48.1]	46.9 [45.6, 48.7]	47.4 [45.2, 48.2]	47.0 [44.7, 48.3]	45.2 [44.4, 48.1]	0.099

Descriptive data are presented as median [interquartile range]. *P*-values represent time-dependent changes within each BMI category and were calculated using the Friedman test

*Pre-OP* preoperative, *M* month, *BMI* body mass index, *ECW* extracellular water, *TBW* total body water

**Table 12:** Changes in SDS phase angle in male and female patients during the course of follow-up

	Pre-OP	3 M	6 M	9 M	12 M	18 M	24 M	*P*-value
Male	-1.85 (-2.35, -1.14)	-2.76 (-3.98, -1.94)	-3.72 (-4.06, -3.31)	-3.31 (-3.89, -2.78)	-3.46 (-4.09, -2.60)	-3.62 (-4.09, -2.44)	-2.29 (-3.48, -1.73)	0.131
Female	0.49 (-0.24, 1.02)	-0.64 (-1.98, 0.46)	-1.34 (-2.14, -0.28)	-1.31 (-1.96, -0.53)	-1.23 (-2.19, -0.39)	-0.69 (-1.75, 0.24)	-0.52 (-1.63, 0.59)	0.003

Descriptive data are presented as median [interquartile range]. P-values represent time-dependent changes within each sex and were calculated using the Friedman test

*Pre-OP* preoperative, *M* month, *SDS* standard deviation score

**Table 13:** Changes in SDS phase angle in patients with BMI < 50 and BMI ≥ 50 during the course of follow-up

	Pre-OP	3 M	6 M	9 M	12 M	18 M	24 M	*P*-value
BMI < 50	0.02 (-1.05, 1.55)	-1.18 (-2.33, 0.05)	-1.88 (-3.40, -0.11)	-1.50 (-2.21, -0.41)	-1.42 (-2.61, 0.04)	-0.64 (-1.67, 0.08)	-0.88 (-2.04, 0.15)	0.018
BMI ≥ 50	-0.55 (-1.31, 0.45)	-1.96 (-2.68, -0.14)	-2.35 (-3.44, -1.71)	-2.39 (-3.13, -1.29)	-2.17 (-3.07, -1.08)	-2.46 (-3.06, -1.29)	-1.63 (-2.87, -0.69)	0.01

Descriptive data are presented as median [interquartile range]. P-values represent time-dependent changes within each BMI group and were calculated using the Friedman test

*Pre-OP* preoperative, *M* month, *BMI* body mass index, *SDS* standard deviation score

**Table 14:** Changes in resting energy expenditure in male and female patients during the course of follow-up

	Pre-OP	3 M	6 M	9 M	12 M	18 M	24 M	*P*-value
Male	2487 (2257, 2853)	2110 (2008, 2364)	2020 (1939, 2219)	1956 (1896, 2122)	1880 (1847, 2041)	1864 (1820, 1945)	1871 (1844, 2012)	0.001
Female	2023 (1906, 2224)	1849 (1680, 2011)	1739 (1576, 1873)	1620 (1532, 1697)	1588 (1506, 1657)	1556 (1435, 1663)	1562 (1476, 1692)	< 0.001

Descriptive data are presented as median [interquartile range]. P-values represent time-dependent changes within each sex and were calculated using the Friedman test

*Pre-OP* preoperative, *M* month, *REE* resting energy expenditure

**Table 15:** Changes in resting energy expenditure in patients stratified by BMI category (< 50 vs. ≥ 50 kg/m²) during the course of follow-up

	Pre-OP	3 M	6 M	9 M	12 M	18 M	24 M	*P*-value
BMI < 50	2001 (1882, 2161)	1832 (1634, 1983)	1725 (1533, 1863)	1655 (1494, 1780)	1612 (1461, 1717)	1601 (1432, 1673)	1577 (1472, 1708)	< 0.001
BMI ≥ 50	2477 (2220, 2775)	2109 (2009, 2318)	2013 (1853, 2178)	1797 (1666, 2092)	1801 (1600, 2046)	1748 (1557, 1939)	1843 (1659, 1999)	< 0.001

Descriptive data are presented as median [interquartile range]. P-values represent time-dependent changes within each BMI category and were calculated using the Friedman test

*Pre-OP* preoperative, *M* month, *BMI* body mass index, *REE* resting energy expenditure

**Table 16:** Changes in bioelectrical impedance parameters during the course of follow-up

	Pre-OP	3 M	6 M	9 M	12 M	18 M	24 M	*P*-value
Absolute fat mass (kg)	67.1 [59.68, 78.63]	52.0 [45.48, 70.60]	43.7 [35.52, 54.34]	37.2 [29.14, 42.23]	33.1 [23.09, 41.34]	30.4 [23.71, 41.03]	32.3 [22.34, 42.84]	< 0.001
Visceral adipose tissue (L)	8.0 [5.61, 10.97]	5.7 [3.72, 8.20]	4.4 [2.61, 6.17]	2.7 [2.04, 4.87]	2.7 [1.50, 3.42]	2.6 [1.39, 3.50]	2.4 [1.48, 3.67]	< 0.001
Fat-free mass (kg)	63.0 [54.88, 73.30]	56.3 [49.55, 63.48]	56.9 [50.43, 63.34]	58.0 [49.78, 62.59]	55.5 [49.98, 65.12]	54.9 [48.38, 63.01]	55.6 [49.51, 63.80]	0.012
Skeletal muscle mass (kg)	31.2 [27.94, 36.43]	27.1 [23.72, 31.40]	26.7 [22.52, 29.57]	26.0 [22.38, 29.41]	25.1 [22.26, 29.64]	25.6 [22.21, 29.33]	27.1 [22.20, 30.09]	< 0.001
Total body water (L)	48.3 [41.79, 55.78]	43.1 [37.76, 48.14]	43.4 [38.40, 48.62]	44.1 [37.45, 47.66]	41.5 [37.70, 48.92]	41.8 [36.67, 47.08]	42.3 [37.43, 47.43]	0.004
ECW/TBW (%)	45.2 [44.43, 46.86]	46.1 [45.17, 47.70]	46.8 [45.34, 48.05]	46.8 [45.45, 47.91]	46.5 [45.28, 47.99]	46.6 [45.41, 47.82]	46.2 [44.82, 47.98]	0.004
Phase angle (°)	5.8 [5.55, 6.40]	5.5 [4.69, 5.88]	5.0 [4.65, 5.37]	5.1 [4.81, 5.40]	5.1 [4.76, 5.52]	5.2 [4.65, 5.90]	5.4 [4.88, 5.76]	< 0.001
SDS phase angle (z-score)	0.0 [-1.13, 0.83]	-1.5 [-2.52, 0.04]	-2.1 [-3.44, -1.11]	-1.6 [-2.91, -1.04]	-1.7 [-2.95, -0.86]	-1.2 [-2.66, 0.00]	-1.0 [-2.23, 0.00]	< 0.001
Phase angle percentile (%)	50.0 [13.00, 80.00]	4.0 [1.00, 52.00]	2.0 [1.00, 13.00]	5.0 [1.00, 15.00]	3.0 [1.00, 16.50]	7.5 [1.00, 49.25]	10.0 [1.00, 43.00]	< 0.001
Resting energy expenditure (kcal/day)	2159.4 [1977.43, 2351.72]	1922.1 [1757.10, 2103.08]	1853.3 [1672.29, 2028.30]	1694.2 [1578.81, 1924.30]	1635.9 [1558.60, 1849.66]	1630.7 [1469.03, 1774.49]	1668.5 [1513.83, 1845.87]	< 0.001

Descriptive data are presented as median [interquartile range]. The p-values for the evaluation of fat mass, fat-free mass, skeletal muscle mass, total body water, resting energy expenditure, ECW/TBW ratio, visceral adipose tissue, phase angle, SDS phase angle, and phase angle percentile were determined using the Kruskal–Wallis test for time-dependent changes

*Pre-OP* preoperative, *M* month, *ECW/TBW* ratio of extracellular water to total body water, *SDS* phase angle, standardized deviation score of phase angle

Absolute reductions were greater in men and in patients with baseline BMI ≥ 50 kg/m². Because these variables are expressed in absolute units, the observed differences are strongly influenced by the higher baseline body size of these patient groups and should therefore not be interpreted as evidence of increased postoperative muscle loss.

Overall, the findings demonstrate that postoperative weight loss was accompanied not only by reductions in adipose tissue but also by decreases in BIA-derived lean body compartments. Stratified trajectories of total body water by sex and baseline BMI are shown in Tables 10 and 11.

### Water compartments

Total body water (TBW) decreased continuously during the postoperative follow-up, paralleling the reduction in body weight and fat-free mass (see Table 5). The largest decrease was observed during the early postoperative period and subsequently reached a plateau, reflecting the stabilization of body composition after the first postoperative year.

In contrast, the extracellular water-to-total body water ratio (ECW/TBW) demonstrated only modest longitudinal changes throughout follow-up (see Table 5). No clinically relevant differences between women and men or between the two BMI groups were observed for ECW/TBW. Small fluctuations were observed at individual follow-up visits but no consistent trend became obvious.

The observed reduction in TBW should be interpreted in conjunction with the changes in fat-free mass because body water represents a major component of lean tissue. Moreover, postoperative alterations in hydration status may influence impedance-derived body composition estimates and therefore should be considered when interpreting longitudinal BIA measurements.

### Phase angle

**Fig. 1:**
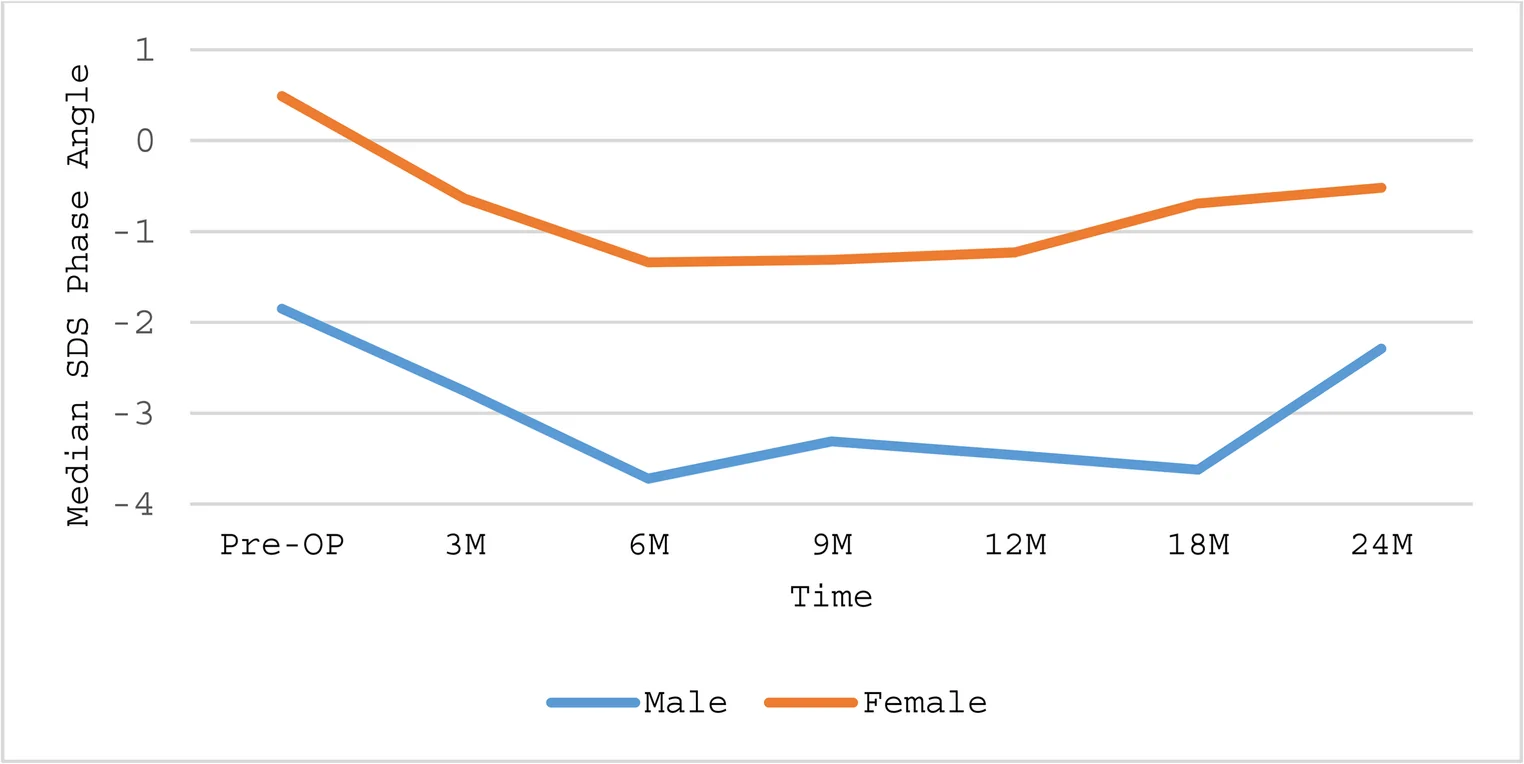
Time course of median SDS phase angle values in male and female patients across the 24-month follow-up period. Values are presented as medians. SDS, standard deviation score; Pre-OP, preoperative; M, month

**Fig. 2:**
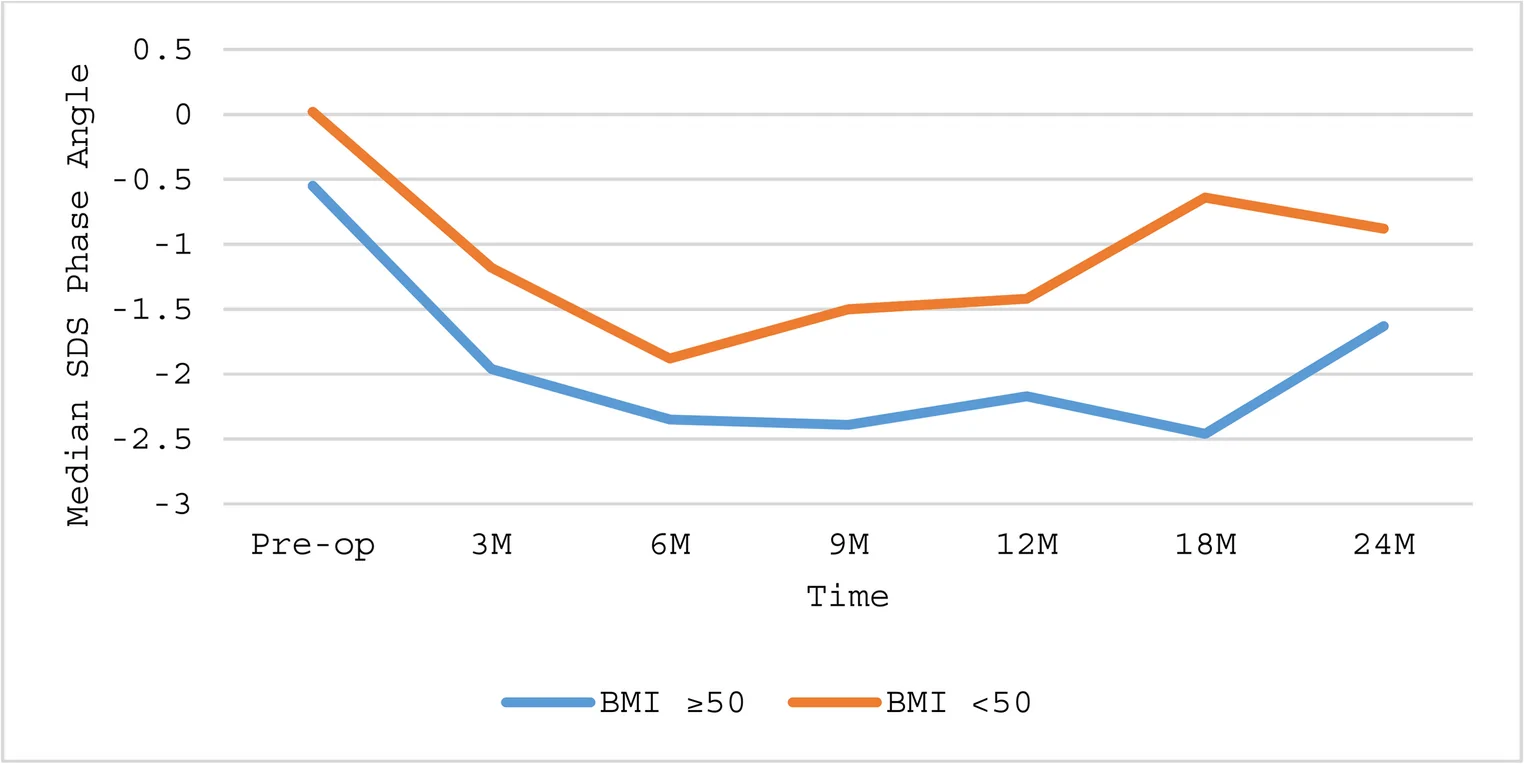
Time course of median SDS phase angle values in patients with BMI < 50 and BMI ≥ 50 across the 24-month follow-up period. Values are presented as medians. SDS, standard deviation score; BMI, body mass index; Pre-OP, preoperative; M, month

Phase angle (PhA) demonstrated a characteristic postoperative pattern throughout the 24-month follow-up (see Figs. 1 and 2). Following sleeve gastrectomy, PhA initially decreased during the first postoperative months before showing a gradual partial recovery during later follow-up. However, postoperative values remained below baseline throughout the observation period. Standardized phase-angle trajectories stratified by sex and baseline BMI are provided in Tables 12 and 13.

This overall temporal pattern was observed in both women and men as well as in both baseline BMI groups. Although descriptive subgroup analyses suggested small differences between groups, no consistent longitudinal divergence of PhA trajectories was evident during follow-up. Therefore, the observed changes primarily reflect an overall postoperative phenomenon within the entire study cohort rather than distinct subgroup-specific responses.

Because PhA is directly derived from resistance and reactance measurements, it is less dependent on body composition prediction equations than variables such as fat mass or skeletal muscle mass. Nevertheless, PhA remains influenced by hydration status, body geometry, age, sex, and measurement conditions. Consequently, the present findings should be interpreted as longitudinal changes in an impedance-derived physiological parameter rather than direct evidence of alterations in cellular integrity or nutritional status. Further studies may provide additional information regarding the clinical significance of postoperative PhA changes.

### Resting energy expenditure

Estimated resting energy expenditure (REE) decreased progressively following SG (see Table 5). The reduction closely paralleled the postoperative decrease in body weight and fat-free mass and was most pronounced during the first postoperative year. Sex- and BMI-stratified REE trajectories are presented in Tables 14 and 15.

Men demonstrated higher estimated REE values than women throughout the observation period, while patients with baseline BMI ≥ 50 kg/m² showed higher estimated REE than those with BMI < 50 kg/m². These findings largely reflect the higher baseline body weight and fat-free mass of these patient groups rather than differences in metabolic adaptation.

Because REE was calculated using the proprietary prediction equations incorporated in the BIA device rather than measured by indirect calorimetry, the reported values should be interpreted as algorithm-derived estimates rather than direct physiological measurements. Consequently, the observed postoperative decrease should not be interpreted as direct evidence of metabolic adaptation but rather as a longitudinal change in the BIA-derived REE estimate.

### Summary of the results

Overall, SG was associated with substantial longitudinal reductions in body weight, BMI, fat mass, visceral adipose tissue estimate, fat-free mass, skeletal muscle mass, total body water, and estimated resting energy expenditure during the 24-month follow-up period (see Tables 3, 4 and 5). The most pronounced changes occurred during the first postoperative year, followed by relative stabilization. Phase angle showed an early postoperative decline with partial recovery over time (see Figs. 1 and 2). Exploratory subgroup analyses suggested differences in absolute body composition trajectories according to sex and baseline BMI; however, these observations should be interpreted cautiously because of baseline differences between groups and the exploratory nature of the analyses. A complementary overview of the longitudinal BIA parameters is provided in Table 16.

## Discussion

The present study investigated longitudinal changes in BIA-derived body composition during the first 24 months following sleeve gastrectomy in patients with class III obesity. The principal findings were a sustained reduction in body weight, fat mass, visceral adipose tissue estimate, fat-free mass, skeletal muscle mass, total body water, and estimated resting energy expenditure. In addition, phase angle demonstrated an early postoperative decline followed by partial recovery during later follow-up. These observations provide a comprehensive overview of postoperative body composition changes beyond conventional anthropometric outcome measures.

While weight loss remains the primary clinical endpoint after bariatric surgery, the present findings demonstrate that substantial alterations also occur in lean tissue compartments and body water distribution. Consequently, assessment of body composition may provide clinically relevant complementary information that cannot be obtained from body weight or BMI alone. Nevertheless, because body composition was assessed exclusively by bioelectrical impedance analysis, the observed changes should be interpreted as longitudinal changes in BIA-derived estimates rather than direct measurements of tissue compartments.

### Changes in body composition after sleeve gastrectomy

The greatest postoperative changes occurred during the first 12 months after surgery, followed by relative stabilization during the second postoperative year. This temporal pattern corresponds well with previous bariatric studies reporting rapid early weight loss followed by a slower stabilization phase [6, 7]. The marked reduction in fat mass represented the major contributor to postoperative weight loss (see Table 4), whereas smaller but clinically relevant decreases were observed for fat-free mass and skeletal muscle mass.

Preservation of lean tissue has become an increasingly important topic in bariatric surgery because excessive reductions in skeletal muscle mass may adversely affect physical function, metabolic health, and long-term weight maintenance [8, 13, 17]. However, the precise clinical importance of the observed lean tissue changes remains difficult to determine in the present cohort. Neither muscle strength, physical performance, protein intake, nor physical activity were assessed. Consequently, the present data cannot determine whether the observed reductions in BIA-derived fat-free mass and skeletal muscle mass were associated with functional impairment.

Our findings are consistent with previous systematic reviews demonstrating that approximately 17,5–31,3% of total postoperative weight loss may originate from lean tissue compartments, particularly during the first postoperative year [13]. However, the reported magnitude of lean tissue loss varies considerably between studies owing to differences in surgical procedure, follow-up duration, nutritional management, exercise participation, and body composition assessment methods. The early PhA decline is also directionally consistent with recent meta-analyses, including a 2026 analysis of 13 studies and 825 patients that reported an overall postoperative reduction in PhA and associations between PhA change and both BMI and FFM change [12, 14].

### Interpretation of BIA-derived body composition estimates

An important aspect of the present study concerns the interpretation of BIA-derived body composition estimates. Bioelectrical impedance analysis represents a practical, non-invasive, and inexpensive method for serial postoperative assessment and is therefore widely used in clinical bariatric practice. However, BIA does not directly measure fat mass, skeletal muscle mass, or visceral adipose tissue. Instead, these variables are estimated using proprietary prediction equations based on electrical impedance measurements together with anthropometric variables.

Several factors may influence the accuracy of these estimates. In particular, hydration status, alterations in extracellular and intracellular water distribution, severe obesity, and rapid postoperative weight loss may affect impedance measurements and consequently influence the calculated body composition parameters. These limitations are particularly relevant during the first postoperative months, when both weight loss and changes in body water compartments are most pronounced.

Published validation studies have demonstrated reasonable agreement between modern multifrequency BIA devices and reference methods such as dual-energy X-ray absorptiometry (DXA) under standardized conditions. Nevertheless, correlation with reference techniques should not be interpreted as complete agreement at the individual patient level [18, 19]. Studies using the seca mBCA 515 also demonstrate that association with DXA does not imply interchangeability at the individual level [20]. Furthermore, BIA-derived estimates of visceral adipose tissue cannot be considered interchangeable with measurements obtained by computed tomography or magnetic resonance imaging [21].

Accordingly, the present findings should be interpreted as longitudinal changes in impedance-derived estimates rather than as direct quantification of fat, skeletal muscle, or visceral adipose tissue. This distinction is particularly important when interpreting relatively small postoperative changes.

### Sex-specific differences

Men exhibited higher baseline body weight, fat-free mass, skeletal muscle mass, visceral adipose tissue estimate, total body water, and estimated resting energy expenditure than women (see Table 1). These findings are consistent with the well-established sexual dimorphism in body composition and fat distribution. Men generally possess greater lean body mass and a higher proportion of visceral adipose tissue, whereas women typically exhibit a larger proportion of subcutaneous fat tissue [15, 16].

Exploratory subgroup analyses suggested larger absolute postoperative reductions in several body composition variables among men. However, these findings should primarily be interpreted in relation to the higher baseline body size of male patients rather than as evidence of fundamentally different biological responses to sleeve gastrectomy. Because of the relatively small number of male participants, the present study was not sufficiently powered to draw definitive conclusions regarding sex-specific postoperative body composition changes.

### Influence of baseline BMI

Patients with a baseline BMI ≥ 50 kg/m² demonstrated higher absolute values for body weight, fat mass, fat-free mass, skeletal muscle mass, visceral adipose tissue estimate, total body water, and estimated resting energy expenditure throughout the study period (see Table 2). These findings are expected because patients with more severe obesity generally possess larger absolute fat and lean tissue compartments before surgery.

The exploratory subgroup analyses further indicated greater absolute reductions in several body composition variables among patients with BMI ≥ 50 kg/m². However, these differences should primarily be interpreted in the context of the markedly higher baseline values rather than as evidence of superior or inferior postoperative treatment response. Relative weight-loss parameters demonstrated a considerably more homogeneous postoperative pattern across BMI categories (see Table 3), suggesting that SG was effective in both patient groups.

Previous studies have reported inconsistent findings regarding the influence of baseline BMI on postoperative outcomes. Baseline BMI is known to influence absolute weight-loss measures, while proportional metrics such as %TWL may attenuate between-group differences [22]. The present findings are generally consistent with this pattern; however, because subgroup analyses were exploratory and based on a limited number of participants, they should be interpreted with appropriate caution.

### Significance of fat-free mass loss

A decline in BIA-derived FFM and SMM was observed, particularly during the rapid weight-loss phase. In the broader bariatric literature, loss of lean tissue is a recognized component of total weight loss, but reported magnitudes vary according to procedure, follow-up interval, measurement method, protein intake, physical activity, and baseline body composition [13, 17, 23]. The present study did not measure dietary intake, protein intake, exercise participation, or adherence to postoperative recommendations and did not assess muscle strength or performance. It therefore cannot determine why FFM estimates changed or whether the change caused functional impairment. Adequate protein intake and resistance-based exercise are supported by external literature as potential countermeasures, but their efficacy cannot be inferred from the current observational data and should be evaluated prospectively [23, 24].

### Phase angle

Phase angle (PhA) represents one of the most interesting BIA-derived parameters because it is calculated directly from electrical resistance and reactance rather than being estimated through prediction equations. In the present study, PhA decreased during the early postoperative period before showing gradual partial recovery during subsequent follow-up (see Figs. 1 and 2). This temporal pattern is in agreement with several previous longitudinal bariatric studies reporting an initial postoperative decline followed by stabilization or partial normalization over time [12, 14].

The physiological mechanisms underlying postoperative PhA changes remain incompletely understood. Several factors may contribute, including rapid reductions in body weight, alterations in intracellular and extracellular water distribution, changes in body cell mass, nutritional adaptation, and postoperative metabolic remodeling. Because these processes occur simultaneously after bariatric surgery, it is unlikely that the observed PhA changes can be attributed to a single biological mechanism.

Importantly, PhA should not be interpreted as a direct marker of cellular integrity or muscle quality. Although lower PhA values have repeatedly been associated with impaired nutritional status, frailty, and adverse clinical outcomes in different patient populations, these associations do not establish causality [25, 26]. Rather, PhA should be regarded as an integrative physiological parameter reflecting the electrical properties of biological tissues within the context of whole-body composition and hydration.

The present study did not include measurements of muscle strength, nutritional intake, inflammatory markers, or physical activity. Consequently, the clinical significance of the observed postoperative PhA changes cannot be fully evaluated. Future prospective studies combining BIA with functional performance tests, nutritional assessments, and imaging techniques may provide further insight into the physiological relevance of longitudinal PhA alterations after bariatric surgery.

### Changes in visceral adipose tissue and water distribution

The decline in the BIA-derived visceral adipose tissue estimate is directionally compatible with the expected reduction in central adiposity after SG [27]. Men had higher baseline estimates, consistent with their greater tendency toward visceral rather than subcutaneous fat accumulation [16]. However, BIA does not directly image visceral or subcutaneous depots, and an MRI comparison in bariatric patients found superior performance of imaging for detecting visceral-fat change [21]. The simultaneous changes in TBW and ECW/TBW further reinforce the need for caution, because postoperative fluid redistribution may influence both the estimated fat-free compartments and PhA.

### Changes in resting energy expenditure

Estimated REE was higher in men and in patients with BMI > = 50 kg/m² at baseline and decreased after surgery. This is consistent with the dependence of prediction equations on body size and FFM. However, REE was not measured by indirect calorimetry, and the study did not assess energy intake or weight regain. The observed reduction should therefore be described as a change in the device-derived REE estimate rather than evidence of metabolic adaptation or a causal determinant of later weight recurrence.

### Clinical implications

The present findings support the concept that postoperative follow-up after bariatric surgery should not be restricted to body weight alone. Although weight loss remains the primary therapeutic objective, simultaneous reductions in fat-free mass and skeletal muscle mass may have implications for physical performance, metabolic health, and long-term weight maintenance. Therefore, assessment of body composition may provide additional clinically relevant information during routine postoperative care.

From a practical perspective, BIA represents an attractive monitoring tool because it is non-invasive, inexpensive, rapid, and easily applicable in the outpatient setting. Repeated BIA measurements may help clinicians identify patients with pronounced reductions in lean tissue or unexpected changes in body water distribution, thereby supporting individualized nutritional counselling and exercise recommendations. Nevertheless, treatment decisions should not rely exclusively on BIA-derived variables but should always be interpreted together with clinical examination, anthropometric measurements, laboratory findings, and functional assessment.

Current evidence supports adequate protein intake and structured resistance exercise following bariatric surgery as strategies to preserve lean tissue and improve muscle strength [17, 23, 24]. Although these variables were not assessed in the present study, our findings further emphasize the need for comprehensive multidisciplinary postoperative care including nutritional support, physical activity counselling, and long-term follow-up. Future studies may clarify whether individualized interventions can further improve postoperative body composition.

### Strengths of the study

Several strengths of the present study should be acknowledged. First, body composition was evaluated longitudinally over a relatively long follow-up period of 24 months using standardized BIA measurements performed according to a uniform institutional protocol. This enabled assessment of postoperative changes beyond the period of rapid early weight loss.

Second, multiple body composition parameters—including fat mass, fat-free mass, skeletal muscle mass, visceral adipose tissue estimate, body water compartments, resting energy expenditure, and phase angle—were evaluated simultaneously, providing a comprehensive overview of postoperative physiological adaptation following sleeve gastrectomy.

Finally, the inclusion of exploratory subgroup analyses according to sex and baseline BMI offers additional information regarding potential differences in postoperative body composition trajectories. Although these analyses were not powered for confirmatory conclusions, they may help generate hypotheses for future prospective investigations.

### Limitations

Several limitations of the present study should be considered when interpreting the findings. First, this investigation was based on a retrospective observational design performed at a single tertiary bariatric center. Consequently, causal relationships cannot be established, and the results may not be fully generalizable to other patient populations or clinical settings.

Second, the overall sample size was relatively small, particularly with respect to the exploratory subgroup analyses according to sex and baseline BMI. Although longitudinal mixed-model analyses allowed inclusion of all available follow-up measurements, the study was not specifically powered to detect subgroup differences. Therefore, both false-positive and false-negative findings cannot be excluded, and subgroup results should be interpreted as hypothesis-generating rather than confirmatory.

Third, no formal adjustment for multiple statistical testing was performed. Because numerous body composition parameters and exploratory subgroup analyses were evaluated, individual statistically significant findings should be interpreted cautiously. Greater emphasis should therefore be placed on the consistency of longitudinal trends than on isolated p-values.

An additional limitation concerns the use of bioelectrical impedance analysis (BIA) as the sole method for body composition assessment. Although modern multifrequency BIA represents a practical and widely accepted technique for longitudinal clinical follow-up, it provides model-based estimates derived from proprietary prediction equations rather than direct measurements of fat mass, skeletal muscle mass, or visceral adipose tissue. Furthermore, BIA-derived estimates may be influenced by hydration status, body geometry, and postoperative changes in fluid distribution, particularly during the rapid weight-loss phase following bariatric surgery. Consequently, the observed changes should be interpreted as longitudinal alterations in BIA-derived body composition estimates rather than direct quantification of individual tissue compartments.

The present study also lacked complementary reference methods such as dual-energy X-ray absorptiometry (DXA), magnetic resonance imaging (MRI), or computed tomography (CT), which would have allowed external validation of the BIA-derived estimates. Likewise, no measurements of muscle strength, physical performance, nutritional intake, inflammatory markers, or objectively assessed physical activity were available. Therefore, the functional and clinical relevance of the observed reductions in fat-free mass, skeletal muscle mass, and phase angle cannot be fully evaluated within the present dataset.

Finally, follow-up was limited to 24 months after surgery. Although this period covers the phase of greatest postoperative body composition change, longer follow-up studies are required to determine whether the observed alterations remain stable or continue to evolve during long-term weight maintenance. Nevertheless, the current follow-up period represents routine clinical practice in many bariatric centers.

## Conclusion

The present study demonstrates that sleeve gastrectomy is associated with substantial longitudinal changes in BIA-derived body composition during the first 24 postoperative months. In addition to the expected and clinically desirable reduction in body weight and fat mass, decreases in fat-free mass, skeletal muscle mass, total body water, and estimated resting energy expenditure were observed, while phase angle showed an early postoperative decline followed by partial recovery.

These findings highlight that postoperative assessment based solely on body weight or BMI may not fully capture the physiological adaptations occurring after bariatric surgery. Incorporating body composition assessment into routine follow-up may therefore provide additional clinically relevant information regarding postoperative changes in lean tissue and body water compartments.

Because all body composition parameters were obtained using bioelectrical impedance analysis, the reported findings should be interpreted as longitudinal changes in impedance-derived estimates rather than direct measurements of individual tissue compartments. Furthermore, the exploratory subgroup analyses according to sex and baseline BMI should be regarded as hypothesis-generating because of the limited sample size and retrospective study design.

Future prospective multicenter studies including larger patient cohorts, standardized nutritional assessment, objectively measured physical activity, functional performance testing, and complementary body composition techniques such as DXA or MRI are warranted to further clarify the clinical significance of postoperative changes in body composition and phase angle following sleeve gastrectomy. Such investigations may help refine postoperative monitoring strategies and optimize individualized long-term patient care.

## Data Availability

Not applicable.
